# Do golf fans walk the talk? Follow-up of spectators’ beliefs and self-reported physical activity 3 months after they attended a professional golf tournament in the UK

**DOI:** 10.1136/bmjsem-2018-000503

**Published:** 2019-01-23

**Authors:** Andrew D Murray, Roger A Hawkes, Paul Kelly, Liz Grant, Nanette Mutrie

**Affiliations:** 1 Physical Activity for Health Research Centre, University of Edinburgh, Edinburgh, UK; 2 Sport and Exercise, University of Edinburgh, Edinburgh, UK; 3 Golf and Health Project, World Golf Foundation, St Augustine, Florida, USA; 4 Medical Department, European Tour Golf, Virginia Water, UK; 5 Global Health Academy, University of Edinburgh, Edinburgh, UK

**Keywords:** physical activity, golf, health, spectating

## Abstract

**Background:**

Previous research of spectators at professional golf tournaments has highlighted that obtaining exercise/physical activity (PA) can be a motivator to attend, and that spectators can engage in health-enhancing PA while at the event. We assessed whether attending a golf event and receiving an intervention improve knowledge and change attitudes related to physical activity, and self-reported physical activity 3 months later.

**Methods:**

Follow-up observational study. Spectators at a European Tour Golf event were given a leaflet about physical activity and health. Three months after that event, we emailed a questionnaire to all 326 spectators who had participated in the original study and provided us their contact details.

**Results:**

135 spectators (41.4%) completed the questionnaire. Among responders, 68.0% ‘agreed/strongly agreed’ that their knowledge relating to PA had increased, 65.1% agreed/strongly agreed that receiving this information at the event made them consider increasing physical activity in daily life and 40.4% reported that they had increased their physical activity during the 3 months after the golf tournament.

**Principal findings/conclusions:**

Golf spectators may contemplate/prepare to increase PA in daily life while a smaller number self-report an increase in PA during the 3 months post intervention at a golf tournament. Spectators’ preferred method for receiving information about ‘active spectating’ is via a big screen. These findings are presented with caution, as respondents may not be representative of all golf spectators.

**What are the new findings?**

- Many golf spectators contemplated, or self-report having actually increased physical activity post receiving an intervention at a professional tournament.
- Public education interventions at golf tournaments may benefit spectators’ individual health and well-being and may contribute to public health and well-being.

**How might findings impact practice in the future?**

- Sporting event organisers can be encouraged to consider public health education and/ or interventions.
- Fans/spectators can receive health benefits, while tournament organisers/ sponsors may find revenue and corporate and social responsibility (CSR) benefits.

## Background

Regular physical activity (PA), and interventions that promote PA, can positively impact longevity, and both physical and mental health.^1–3^ The 2013 economic burden of physical inactivity to health globally was conservatively estimated at US$53.8 billion.^4^ A clear aim for practitioners, policy-makers and indeed researchers is to support and influence more people to be more active more often. Global targets to reduce physical inactivity by 10% by 2025 and 15% by 2030 have been set.^5^

There is no single or simple solution to the complex problem of physical inactivity. Best investments to increase levels of PA have been described, working in partnership and across sectors that include (1) communication and public education, (2) urban design and infrastructure, (3) sport and recreation, and (4) community programmes.^5–7^

One initiative that may contribute to addressing physical inactivity is using golf tournaments. Professional golf tournaments draw over 10 million spectators per year.^8^ The best available evidence suggests that golf spectators report ‘exercise/physical activity’ and ‘potential health benefits gained’ as considerations in attending professional golf tournaments.^9–13^ In golf, unlike many other sports where spectators are seated, spectators often walk portions of the course when watching the action.

Collaborations between The European Tour, The R&A, The World Golf Foundation and academic institutions have been established to promote PA at golf tournaments which include some of the biggest male (the Open Championship, the Ryder Cup) and female (Women’s British Open) golf events globally. Our previous research has highlighted that tournament spectators can gain health-enhancing PA. Our previous study showed that 84.7% of participating spectators achieved their daily recommended PA, when measured by step count, while spectating.^12^

However, the overall public health benefit of a single day of spectating even for 10 million persons is modest. Gaps in knowledge exist around:

1. Whether the spectating experience could be used to influence knowledge, perceptions and attitudes regarding PA beyond the tournament;
2. Whether the spectating experience could be used as a ‘teachable moment’ to increase PA in spectators beyond their attendance at a tournament;
3. Preferred methods for providing information during the tournament.

We aimed to contribute to those knowledge gaps through this follow-up study. Our research questions were:

1. How do spectators report knowledge, perceptions and attitudes regarding PA 3 months after attending a golf event and providing baseline data?
2. Do spectators report that the event influenced levels of PA 3 months after the golf tournament?
3. What methods of providing information and encouraging behaviour change do spectators report favouring?

## Methods

Approximately 600 persons out of 1500 paying spectators attending the European Tour Paul Lawrie Matchplay event, Scotland, UK (4–7 August 2016) were provided with information relating to spectating and health in written form having been approached by trained researchers at random.

The inclusion and exclusion criteria, detailed in table 1, were used to determine suitability.

**Table 1:** Inclusion and exclusion criteria

Inclusion criteria	Exclusion criteria
Spectators at the European Tour Paul Lawrie Matchplay Aged 18 or over Able to walk (walking aids permitted) Unstable cardiovascular disease not reported Able to provide email address	Non-spectators (eg, staff, marshalls, players, caddies) Aged under 18 years Inability to walk Reported unstable cardiovascular disease (critical aortic stenosis, unstable angina, myocardial infarction within 6 weeks—a medical doctor was part of the research team and could provide individual case advice) Email address not provided

Following consent, 339 spectators completed a questionnaire. The participant was offered the opportunity to wear a pedometer and then spectated in a manner of their choosing. Prior to exiting the venue, participants returned the pedometer to a member of the research team who checked and recorded the number of steps taken, and the time returned. These step count data and reasons for attendance data have previously been published.^12^

Three months following the event, a questionnaire was emailed via Google forms (Google, Mountain View, California, USA) to each individual who had provided consent and an email address (n=326). A reminder email was sent 2 weeks later. The questionnaire was developed from studies assessing PA knowledge in other population groups,^14 15^ using key concepts from Prochaska and DiClemente’s stages of change model,^16–18^ and following author discussion.

## Results

Emails requesting completion of the questionnaire was sent to 326 persons, of which 11 were rejected by the server. In total, 135 out of a potential 326 participants returned the questionnaire representing 41.4% of those eligible. Of those 135 completing the survey, 129–131 responses were received for each question.

Responses of the 135 respondents on a five-point Likert scale are shown in table 2 and are summarised below.

**Table 2:** Perceptions, attitudes and behaviours regarding physical activity, and relation to spectating at Paul Lawrie Matchplay

Item	Number of responses	% Strongly agree	% Agree	% Neither agree nor disagree	% Disagree	% Strongly disagree
Walking while spectating at golf events is likely to be beneficial for health. How much do you agree with this statement?	131	61.8	35.9	0.8	1.5	0.0
Receiving information at the Paul Lawrie European Tour event about the benefits of walking/physical activity helped increased my knowledge in this area. How much do you agree with this statement?	131	21.4	46.6	22.9	7.6	1.5
Receiving this information will make me consider being more physically active at golf tournaments. How much do you agree with this statement?	129	17.8	41.1	33.3	7.0	0.8
Receiving this information has made me consider being more physically active in daily life. How much do you agree with this statement?	129	17.8	47.3	25.6	7.8	1.6
I have done more physical activity (including walking) since spectating at the Paul Lawrie golf tournament. How much do you agree with this statement?	131	7.6	32.8	38.2	16.8	4.6
Being provided with information about potential health benefits of spectating make it more likely I will attend a golf tournament. How much do you agree with this statement?	130	12.3	24.6	46.2	16.2	0.8

- 68.0% (n=131) agreed or strongly agreed that receiving information at the Paul Lawrie European Tour event about the benefits of walking/PA helped increase their knowledge in this area.
- 58.9% (n=129) agreed/strongly agreed that receiving information will make them consider being more physically active at golf tournaments.
- 65.1% (n=129) agreed/strongly agreed that receiving information at the tournament will make them consider being more active in everyday life.
- 40.4% (n=131) agreed/strongly agreed that they had done more PA (including walking) since spectating at the Paul Lawrie golf tournament.
- 36.9% (n=130) agreed or strongly agreed that having been provided with information about potential health benefits of spectating make it more likely they will attend a golf tournament.

**Figure 1:**
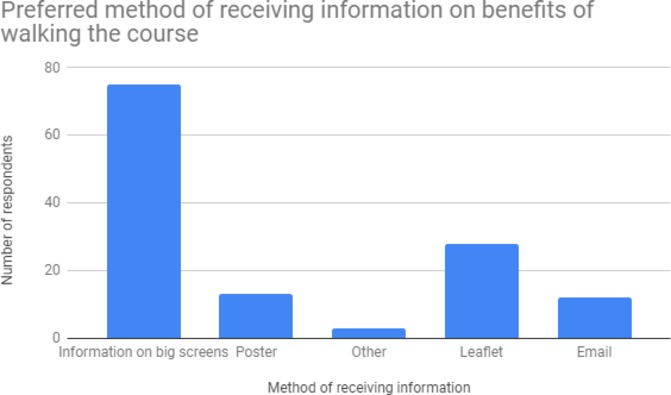
Preferred method of providing information to golf spectators on the benefits of walking the course.

When asked as to the most useful ways to give golf spectators information about the benefits of walking the course, information provided on the big screens constructed around the course was the most popular option. Further detail is shown in figure 1.

## Discussion

### Principal findings

This study showed that it was feasible to collect follow-up data related to PA for health from a sample of golf tournament spectators. Results showed positive self-reported views on the usefulness and impact of receiving PA for health information at golf tournaments.

#### Feasibility and generalisability

Obtaining follow-up data at 3 months via a questionnaire for spectators attending golf tournaments is feasible—the response rate was 41.4%. Participants who responded to the survey may or may not be typical/representative of golf spectators, so the results should be interpreted accordingly.

#### Influencing knowledge, PA levels, behaviours and attitudes

A clear majority of respondents (68.0%) agreed or strongly agreed that information received at this golf tournament had helped increase their knowledge of PA for health. Almost two-thirds indicated that receiving information relating to active spectating had influenced them to consider becoming more active in everyday life.

Around two-fifths of respondents indicated that they had increased their physical activity levels at least 3 months post intervention. This increase was self-reported as opposed to measured by pedometer to maximise participation and minimise participant and research team burden.

#### Providing PA information to spectators

Some spectators (36.9%) agreed or strongly agreed that being provided with information about potential health benefits of active spectating would make it more likely that they will attend future golf tournaments. This offers an additional attractive marketing angle for tournament promoters. Spectators’ preferred method for receiving this information was via the big screens at golf events, followed by leaflet, with poster and email communication less popular.

### Comparison with the literature and explanation for findings

There is little previous research regarding the provision of information on PA to golf spectators, their attitudes towards PA^9–13^ and interventions to influence PA levels. There is no research following up any intervention regarding PA and golf spectators. Thus, comparisons will be made with a broader evidence base.

#### Knowledge and attitudes relating to PA for health

Previous research from this golf spectator cohort discovered that they have high habitual levels of PA compared with the general population.^12^ We speculate that many golf spectators are likely to be golfers themselves and thus are more likely to be regularly physically active. Spectating at golf tournaments does offer spectators the opportunity to move around and follow play from a variety of locations. We could not identify studies of cohorts of spectators from other sports and their knowledge/habits relating to PA.

Public education and communication are recognised as an investment that works in increasing PA.^6 7^ Prochaska and DiClemente’s transtheoretical/stages of change model was originally developed for smoking cessation and has been supported and adapted for sport/PA.^18 19^ Being underinformed on potential consequences of a health behaviour may lessen the chances of positive behaviour change.^20^ Providing information via for example big screens, in event programmes or in leaflet form at golf events can be considered public education.

Providing education can help shift decisional balance in favour of progressing positively along the stages of change.^18 21^ It is likely that if education/intervention is provided by persons or in a context that persons identify with/admire, then it may be well received.^22 23^ Football Fans in Training leveraged players, club and sporting club identities to influence education and behaviour change.^22^ Our study engaged with professional golf players (tournament host and previous major champion Paul Lawrie) to provide key messages in the literature provided. Sixty-eight per cent of respondents reported that information received at the golf tournament had helped increase their knowledge in this area. While they may not be fully representative of all spectators at this and other golf tournaments, it would appear that some spectators can be positively engaged in learning about PA for health.

#### Influencing PA levels

In total, 65.1% of our sample indicated that receiving information relating to active spectating had influenced them to consider being more active in everyday life. Even for those meeting the minimum guidelines for PA, greater health and well-being benefits can be gained by doing more, for most people. Using Prochaska and Di Clemente’s stages of change/trans-theoretical model,^16 17 20^ these persons can be represented as being in the ‘contemplative’, ‘preparation’ or ‘action’ phase when surveyed.

Over two-fifths (40.4%) of respondents in the present study self-report positive behaviour change in increasing PA at 3 months post intervention. The magnitude of increase was not assessed so as to maximise participation (minimise participant burden). These persons can be represented as being in the ‘action’ phase when surveyed. Maintaining improved PA levels would provide longevity, physical and mental health benefits to people and populations.

With many spectators contemplating, preparing to take action, or taking action to increase PA, interventions at tournaments have the potential to be a teachable moment aimed to move participants further along the cycle of change, and positively influence PA knowledge, and achieved PA. We underscore that it is not clear whether the sample who responded to our survey are representative of other spectators at this, or other golf events. Nevertheless, there is an important proportion of golf spectators whose knowledge and behaviours can be positively influenced.

#### Providing PA information and intervention to spectators

To increase PA generally,^5 6^ and more specifically to achieve a legacy after a major sporting event,^23 24^ strategy and collaboration are required. A meta-analytic review of tailored health behaviour change interventions shows that tailoring for the audience, to theoretical concepts of the stages of change and to context is important.^25^ Collaboration to assist tailoring and delivery for this initiative was achieved with a walking charity (Paths for All), academics (University of Edinburgh), tournament promoters (the European Tour, 4Sports), professional golf players, and local and national policy-makers (East Lothian, the Scottish Government, the (UK) All-Party Parliamentary Group on Golf), which likely contributed to the success of the intervention and can assist further delivery at future tournaments.

#### Co-benefits for tournament promoters and methods of information provision

Over a third of spectators (36.9%) agreed or strongly agreed that being provided with information about potential health benefits of active spectating make it more likely that they will attend future golf tournaments. This offers an additional attractive marketing angle for tournament promoters aimed at increasing spectator volume (and revenue) and engagement. This is in keeping with other studies suggesting more priority could be given to promoting exercise/PA benefits of attending golf events.^9 10 12^ Spectators’ preferred method for receiving this information is via the big screens, followed by leaflet. These are both common methods for sharing information at golf and other major sporting events. Both these methods likely offer value, as printed material can be tailored to appeal across a range of stages of change, while spectators welcome the engagement of the big screen/billboard.

### Implications and recommendations for research, policy and practice

Studies of spectator populations at other tournaments are likely to be influenced by factors including, but not limited to, cultural factors, type of tournament, engagement of player ambassadors and local facilities for PA. Thus, while further larger research studies may seem attractive, small-scale implementation followed by pragmatic evaluation and the implementation of improvement and, if appropriate, scale-up science may offer more value. Broad principles will apply, but the detail is likely to be different based on many factors and a pragmatic approach may be best.

Governing bodies for sport, golf promoters and marketeers can be encouraged to collaborate with leading players, PA experts and a range of other stakeholders to encourage practices and policies that promote walking at the event,^26 27^ but also using the ‘teachable moment’ of attendance at tournaments to impact knowledge around physical activity and encourage positive health behaviour change. This could improve public health for fans and communities, and need not be limited to increasing PA, but also support healthy eating, sustainable transport, wearing sunscreen and so on.

Golf spectating does offer an opportunity for PA in this particular setting and population. Attendance can be encouraged, and spectators can actively be supported to engage in PA through promotional efforts ahead of and during each professional golf event.^26^ Fans/spectators can receive public health benefits, while tournament organisers/sponsors may find revenue and corporate and social responsibility benefits. Collaborations thus far have produced interventions at leading tournaments worldwide including the Ryder Cup, the Open Championship, The Women’s British Open, the Shenzhen Open (China), Indonesia Open and Andalucía Masters (Spain) among others. Potential benefits of promoting PA for spectators at events are shown in table 3.

**Table 3:** Potential benefits of promoting physical activity for spectators at events

Members of public/spectators	Tournament promoters	Government/local authority
Individual health and well-being	Increased volume of spectators—revenue Corporate and social responsibility	Legacy from event Public health and well-being

### Strengths and limitations

This study was pragmatic in its approach.

Strengths include building on the existing literature and frameworks to describe an innovative approach to engage with spectators at a major sporting event. Those study participants reported an increased awareness of PA benefits, and in some cases increases in reported PA levels. Although further study is required, we discovered that attendance at a golf tournament could present a teachable moment, and that there are potential untapped public health benefits related to major sporting events. This study demonstrated that over 40% of those eligible returned completed questionnaires via email and that conducting research following up spectators from sporting events is feasible. It also describes the value of co-production/collaboration which will support improvement of interventions and scale-up where relevant.

Limitations are apparent. While all those who agreed to take part were emailed and sent a reminder, those who completed the survey may be a unique subset, potentially more interested in PA than those who did not return correspondence leading to potential selection bias. Individuals were aware that they were taking part in a study and may have changed their behaviours recognising they were being observed (Hawthorne effect). Twenty-four persons supplied email addresses that failed to deliver or were illegible, while an unknown number may have ended up in ‘spam’ folders. While the tournament took place in August, follow-up was in October onwards, when weather conditions and other factors may affect people’s motivation and ability to achieve PA. No objective measures of PA levels beyond the event were captured. The sample size and other limitations limit generalisability, particularly recognising the worldwide distribution of golf tournaments and significant differences in culture/context.

## Conclusions

Post intervention, many golf spectators contemplated increasing PA in daily life while close to half of this sample self-report having actually increased PA. Spectators’ preferred method for receiving information on PA benefits, and how this can be achieved is via the big screens erected at major tournaments.

Public education interventions at golf tournaments may benefit spectators’ individual health and well-being, while local authorities may see a contribution to public health and well-being. Tournament promoters may attract an increased volume of spectators, while interventions can help achieve company corporate and social responsibilities.

These data are presented with caution, recognising that while the findings are novel and point to potential exciting opportunities to deliver to the WHO’s agenda of increasing PA, those responding to the survey may not be fully representative of spectators at golf tournaments.
